# Protein Adductomics: Analytical Developments and Applications in Human Biomonitoring

**DOI:** 10.3390/toxics7020029

**Published:** 2019-05-25

**Authors:** George W. Preston, David H. Phillips

**Affiliations:** Environmental Research Group, Department of Analytical, Environmental and Forensic Science, School of Population Health and Environmental Sciences, King’s College London, Franklin-Wilkins Building, 150 Stamford Street, London SE1 9NH, UK; george.preston@kcl.ac.uk

**Keywords:** haemoglobin, albumin, mass spectrometry, biomarkers, protein adducts

## Abstract

Proteins contain many sites that are subject to modification by electrophiles. Detection and characterisation of these modifications can give insights into environmental agents and endogenous processes that may be contributing factors to chronic human diseases. An untargeted approach, utilising mass spectrometry to detect modified amino acids or peptides, has been applied to blood proteins haemoglobin and albumin, focusing in particular on the *N*-terminal valine residue of haemoglobin and the cysteine-34 residue in albumin. Technical developments to firstly detect simultaneously multiple adducts at these sites and then subsequently to identify them are reviewed here. Recent studies in which the methods have been applied to biomonitoring human exposure to environmental toxicants are described. With advances in sensitivity, high-throughput handling of samples and robust quality control, these methods have considerable potential for identifying causes of human chronic disease and of identifying individuals at risk.

## 1. The Exposome and Adductomics

Many decades of epidemiological observations have indicated that incidences of chronic human diseases are likely to result from a combination of environmental exposures to chemical and physical stressors, and predispositions inherent in human genetics. The wide geographical variation of many such diseases implies that it is environmental factors that play the dominant role, and not inherited predisposition, in disease causation [1], but knowledge of what the environmental factors are is often far from complete. As a consequence, estimations of overall risks associated with these factors are inaccurate and important associations may go undetected. These limitations have recently been framed within the context of the *exposome*, which can be thought of as the environmental counterpart of the genome. Conceptually, the exposome aims to reflect the totality of environmental exposures throughout the human lifespan, and to take into account both external components (e.g., exogenous environmental agents) and internal ones (e.g., endogenous cellular processes that give rise to altered stasis or function) [2,3,4,5]. For strategies to improve human health to be effective, it is essential to unravel the causes of chronic human diseases and to assess accurately their risks. The goal of studying the exposome (i.e., of *exposomics*) is disease prevention through the acquisition of a broad scientific perspective that encompasses health, environmental, educational, socioeconomic and political factors [6,7,8,9].

Two recent collaborative projects have applied the exposome concept to investigating environmental impacts on human health by assessing environmental exposure at personal and population levels within existing short- and long-term population studies. In the *EXPOsOMICS* project the emphasis has been on the measurement and impact of air and water pollution, studied in a number of adult and child study populations [10]. In the *HELIX* project the focus has been on early-life events, examining exposure to a range of chemicals and physical agents in existing birth cohorts [11]. Both these projects utilised a combination of exposure monitoring, using mobile and static monitors, smartphone and satellite data, and omics techniques to investigate biomarkers associated with exposures. The multi-omic approach has included metabolome, proteome, transcriptome, epigenome and adductome profiles. While many of the results of these interrelated analyses have yet to emerge, it is anticipated that new insights into the importance of environmental factors in the aetiology of human diseases will ensue and that the studies will point the way to improved strategies for monitoring human exposures and their health consequences.

While these projects have focused on human exposures and health outcomes, broader ecological issues may also be addressed by the exposome concept. The adverse outcome pathway (AOP) concept seeks to define the initial molecular events that culminate in adverse (toxicological) endpoints [12]. There is currently much discussion of how to assess the properties of complex mixtures of chemicals, taking into consideration possible positive and negative interactions between their components, in order to refine hazard identification and risk assessment. It has been proposed that considering the relative contributions of components of the exposome in relation to complex mixtures combined with a mechanistic understanding of the induced adverse effects, may improve the integrated risk assessment for both human and environmental health [13]. 

Electrophiles have long been suspected in the causality of cancer and other chronic diseases. Because they are reactive, they can be measured indirectly through the adducts they form with protein and DNA. Indeed, damage to, or modification of, DNA by reactive intermediates of chemical carcinogens or by ionising and non-ionising radiation is a key early event in the carcinogenic process. The exposome concept encompasses a “top-down” approach to identifying environmental factors that determine susceptibility to disease throughout the entire lifespan. In parallel, a “bottom-up” approach can investigate biomarkers specific for certain environmental exposures, based on knowledge of environmental carcinogens and their pathways of metabolic activation. As part of this approach, protein adductomics constitute the untargeted investigation of modification of proteins by endogenous or exogenous agents.

## 2. Approaches to Protein Adductomics

The concept of an adductome (that is, a collection of additional products) implicates two types of reactant: Those that add, and those to which are added. In the context of the present discussion, these are nucleophilic protein sites (amino acid residues) and electrophilic toxicants, respectively^1^. Reactants of either type are potentially diverse, meaning that the adductome could be vast. From an analytical standpoint, this potential vastness (i.e., structural diversity) is problematic because of the lack of a common ‘handle’ or ‘signature’ by which to purify and identify the adducts. Accordingly, investigators have focused on adducts of either specific nucleophiles or specific electrophiles. If the investigator’s aim is to discover biomarkers of exposure, a nucleophile is selected and the electrophiles to which it adds are captured; if the aim is instead to discover targets, an electrophile is selected and the nucleophiles that add to it are captured. Given that the focus of this review is on biomarkers of environmental exposure, we will concentrate on the former approach. The latter approach is also important, however, because it is a route by which novel adducts could be accessed, either directly [14,15] or indirectly [16].

Of the methods that capture electrophiles, the most advanced methods are based on haemoglobin (Hb) and human serum albumin (HSA). There have been a number of important methodological developments since Rappaport et al. reviewed the subject in 2012 [17]. Another, related review [18] was published during the preparation of the present review.

### 2.1. Hb as A Target of Electrophiles

Hb is found in the erythrocytes, where it functions as an oxygen carrier. Its high concentration and reactivity (see below) make it a likely target of electrophiles, and its long lifetime in vivo (126 days, the lifetime of an erythrocyte [19]) presumably gives the resulting adducts an opportunity to accumulate. Human Hb A, the major form of Hb in adults, is a tetramer composed of two α-chains and two β-chains. The four chains, each of which binds one molecule of haem, all adopt similar folds in the tetramer. The α- and β-chains have several amino acid residues in common, including the *N*-terminal valine residues [20]. The α-amino groups of these terminal residues are nucleophilic, and have been observed to react with toxicologically-relevant electrophiles [21]. The *N*-terminal α-amino groups of the α- and β-chains have similar pK_a_ values and similar reactivity towards certain electrophiles (e.g., the acetylating agent acetic anhydride), but not necessarily towards all electrophiles [22]. For example, another acetylating agent, methyl acetyl phosphate, has been observed to modify the *N*-terminus of only the β-chain [23]. 

The β-chain of Hb possesses a cysteine residue (Cys-β93) for which there is no equivalent in the α-chain [20]. Adducts of Hb Cys-β93 have been the subject of both targeted and, to a lesser extent, untargeted adductomic analyses (see below). A targeted adductomic method (i.e., a method involving simultaneous monitoring of multiple known/hypothesised adducts) was used to monitor Hb adducts of 15 different aromatic amines (e.g., 4-aminobiphenyl) in tobacco smokers’ blood [24]. These, it should be pointed out, are not adducts of the amines themselves, but rather of the corresponding arylnitroso compounds [25]. Arylnitroso compounds form via oxidation of the amines’ *N*-hydroxy metabolites, in a reaction for which, in the erythrocyte at least, the oxidant is the oxy form of Hb itself. The Cys-β93 adducts of arylnitroso compounds are *N*-arylsulfinamides, which hydrolyse under acidic conditions to regenerate their corresponding aromatic amines [26]. On this basis, detection of the aromatic amines liberated by acid hydrolysis of *N*-arylsulfinamides has been used as an indirect way of detecting the adducts [24].

### 2.2. The N-alkyl Edman Method

The analytical tractability of Hb *N*-terminal adducts is due to a general property of *N*-terminal amino acid residues, namely their ability to be detached from the rest of the protein via Edman degradation. This is a procedure that was originally developed for protein sequencing, but which was modified in the 1980s by Ehrenberg and co-workers for the analysis of Hb *N*-terminal adducts [27]. Ehrenberg and co-workers’ procedure has been referred to as the ‘*N*-alkyl Edman method’ because of its ability to detect, for example, *N*_α_-methyl and *N*_α_-ethyl substituents [28,29]. In fact, the observed *N*_α_-substituents have not been limited to simple alkyl groups, but for convenience the modified *N*-terminal amino acid is referred to as *N*-alkylvaline. Edman’s original procedure involved reacting the α-amino group of a peptide with phenyl isothiocyanate, which rendered an acid-labile product [30]. Treatment of this product with anhydrous acid liberates the terminal amino acid as an anilinothiazolinone, which is then isomerised in aqueous acid to a phenylthiohydantoin (PTH) [31]. Ehrenberg and co-workers found that Hb with *N*-terminal *N*-alkylvaline (i.e., a secondary amine) reacted with isothiocyanate reagents in the same way as unmodified Hb, but that the resulting derivatives were labile even under neutral conditions [27]. The final product, a substituted PTH, could therefore be isolated using conditions under which unmodified Hb remained intact.

In subsequent iterations of the *N*-alkyl Edman method, the isothiocyanate reagent was varied so as to generate analytes appropriate for particular analytical methods. The most recent iteration, the ‘FI*R*E procedure’, uses fluorescein isothiocyanate (‘FI*R*E’ being a contraction of ‘fluorescein isothiocyanate’, ‘*R*-group’ and ‘Edman degradation’) [32]. The FI*R*E procedure was initially developed with targeted analysis in mind, but was later adapted for untargeted analyses (‘FI*R*E screening procedure’ [28]).

### 2.3. The Role of Tandem Mass Spectrometry in Protein Adductomics

Like most other adductomic methodologies, the FI*R*E screening procedure utilises tandem mass spectrometry (MS/MS) for the detection of adducts. MS/MS, as its name suggests, involves two stages of mass analysis. The first stage is for intact precursor ions (e.g., protonated molecules) and the second stage is for product ions (i.e., fragments of precursor ions). A process of fragmentation takes place in between the two stages. Mass analysis can be performed in either a static mode, whereby ions of specified mass-to-charge ratio (*m*/*z*) are isolated, or a dynamic mode, whereby a continuous range of *m*/*z* values is scanned. Either stage can be performed in either mode, meaning that a number of different types of experiment are possible. In selected reaction monitoring (SRM), a technique commonly used for targeted analyses, ions of pre-specified *m*/*z* are isolated at both stages. Isolation is achieved by defining a narrow window of permissible *m*/*z* values and is often done using a quadrupole mass filter. An apparatus commonly used for SRM is the triple quadrupole mass spectrometer, which consists of two quadrupole mass filters, with a collision cell between them, connected in series. The first and second stages of mass analysis take place in the first and second filters, respectively, with fragmentation taking place in the collision cell. Other MS/MS techniques of relevance to this review are precursor ion scanning, data-dependent acquisition (DDA) and data-independent acquisition (DIA). These will be covered in more detail in the sections concerning HSA adductomics.

### 2.4. Stepped MS/MS Methods 

Several adductomic studies have employed stepped methods, which can be thought of as hybrids of SRM and scanning. A stepped method consists of a sequence of SRM experiments that collectively resemble a scan. In considering how the methods work, it is instructive to think of adducts’ structures in terms of two distinct parts: A constant part that derives from the nucleophile (common to all precursor ions) and a variable part that derives from the electrophile (variable among precursor ions). It follows, therefore, that a given product ion (or neutral fragment) will be either constant or variable depending on how the precursor ion becomes broken up into fragments. Given that the variable parts of the precursor ions are unlikely to be known *a priori*, the constituent SRM experiments of a stepped method must be necessarily arbitrary. For this reason, it is common to see lists of equally-spaced integer or half-integer *m*/*z* values [28]. We have referred to these arbitrary values as sampling points [33]. The idea of an arbitrary SRM experiment might strike the reader as odd, since SRM is traditionally used for targeted analyses, but for untargeted analyses it does not matter where the sampling points fall. The important thing is that, collectively, they are able to capture all relevant adducts. The limitation of stepped methods is their low resolution, which means that they are unable to identify adducts unambiguously purely on the basis of mass. Their value, therefore, tends to be in providing a quantitative description of the distribution of adducts.

### 2.5. The FIRE Screening Procedure

**Figure 1:**
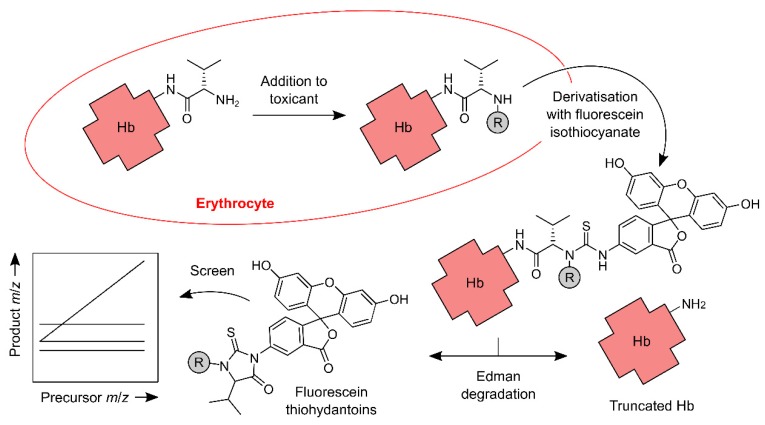
Main steps of the FI*R*E (‘fluorescein isothiocyanate’, ‘*R*-group’ and ‘Edman degradation’) screening procedure for Hb *N*-terminal adductomics. The procedure detects ‘R’ groups, which are generated when an *N*-terminus of Hb reacts with an electrophile in vivo. The *N*-termini are derivatised with fluorescein isothiocyanate, and derivatives with ‘R’ groups are selectively decomposed to the corresponding fluorescein thiohydantoins. The thiohydantoins are analysed using LC and online ‘stepped’ triple quadrupole mass spectrometry.

The FI*R*E screening procedure [28] is a method for untargeted detection of Hb adducts (Figure 1). It is a stepped method akin to the ‘adductome approach to detect DNA damage’ developed by Kanaly et al. [34]. In the FI*R*E screening procedure, different precursor ions (protonated fluorescein thiohydantoins, FTHs) are captured at the first stage of mass analysis via one of 136 different windows. Each window is approximately 0.7 *m*/*z* units wide, and the *m*/*z* values on which the windows are centred are 1 Da apart. Thus, by cycling through all 136 windows, the method can capture a wide range of precursor ions and can, therefore, detect the corresponding range of mass shifts (between +14 and +149 Da). Once captured, a precursor ion is fragmented, and its products are passed to the second stage of mass analysis. Here, a set of fixed windows permit only constant product ions to pass to the detector (implicates loss of variable neutral fragments), and a variable window permits only variable product ions to pass (implicates loss of constant neutral fragments). If the right combination of constant and variable product ions is detected, then the presence of a corresponding FTH, and therefore Hb adduct, can be inferred. This MS/MS is done ‘online’ following the chromatographic separation of the FTHs, and the data thus generated are, like those reported by Kanaly et al., visualised as an ‘adductome map’, usually a plot of *m*/*z* against retention time [28,34,35].

Carlsson et al. used their procedure to screen the blood of smokers and non-smokers and detected 26 features of interest; this study is described below in Section 3. 

### 2.6. HSA as A Target of Electrophiles

HSA is the major protein in human plasma. Its lifetime in vivo, whilst shorter than that of Hb, is presumably still long enough for adducts to accumulate. In vivo, HSA binds fatty acids, scavenges metal ions, and contributes to the oncotic pressure of blood [22]. Extensive use of HSA has been made for the biological monitoring of toxicants, and a detailed account of this can be found in the recent review by Sabbioni and Turesky [36]. For the purposes of the present review, we focus on providing a background to the untargeted HSA adductomics studies.

Thus far, HSA contains a number of nucleophilic sites, including (but not limited to) histidine residues, lysine residues and a single reduced cysteine residue (Cys-34). Notably, histidine residues in HSA, as in Hb, are targets of epoxides [37,38]. Lysine residues in serum albumins are notable targets of aflatoxin B_1_ dialdehyde [39,40]. 

Cys-34 is the only site in HSA for which untargeted adductomic methods have been developed. The motivation to look at this particular site is related to the unique chemistry of thiol groups, and the fact that HSA Cys-34 accounts for the majority of such groups in human plasma [41]. Given that the reacting species is a thiolate anion rather than a thiol group proper [41], adduct formation should be promoted by alkaline conditions and/or basic groups within the local protein environment. The pK_a_ of the HSA Cys-34 thiol group is controversial, but is generally regarded to be lower than that of a typical thiol group [41]. In the three-dimensional structure of HSA, as determined by X-ray crystallography, the side chain of Cys-34 is partially buried [42]. On this basis, it has been inferred that there might be a limit to the size of the electrophiles that HSA Cys-34 can add to. It has also been recognised, however, that the tertiary structure of HSA is dynamic and that Cys-34 may become less buried upon deprotonation of the thiol group [17,43]. HSA Cys-34 is reactive towards a variety of toxicologically-relevant electrophiles, including sulphur mustard and metabolites of aromatic amines [44,45], and can also undergo oxidative transformations [46,47]. It appears that, in vivo, a substantial proportion of the HSA Cys-34 thiol groups is *S*-thiolated (*S*-[cystein-*S*-yl], *S*-[glutathion-*S*-yl] and so on), and a smaller, but appreciable proportion is found as the corresponding sulfenic, sulfinic or sulfonic acids [41,47].

### 2.7. HSA Cys-34 Adductomics 

**Figure 2:**
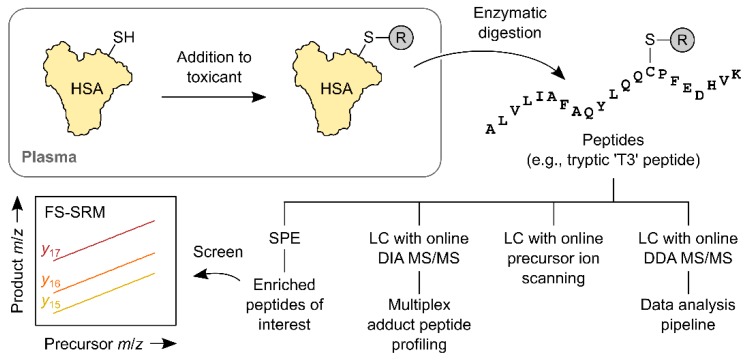
Main steps of published HSA Cys-34 adductomic workflows. The reaction of HSA with an electrophile in the blood plasma installs an ‘R’ group at the Cys-34 site. The HSA is isolated from plasma or serum and digested—usually with trypsin—to produce a mixture of peptides. Some of the peptides contain ‘R’ groups and others do not (the introduction of an enrichment step prior to digestion can limit the number of those that do not). Peptides are then separated chromatographically and analysed using MS/MS. One of the MS/MS methods, a stepped triple-quadrupole method termed FS-SRM, is depicted. This method monitors three variable product ions of the tryptic ‘T3’ peptide (*y*_15_, *y*_16_ and *y*_17_).

To date, methods for HSA Cys-34 adductomics have been based exclusively on peptide analytes (Figure 2). When HSA is digested with trypsin, and no cleavages are missed, Cys-34 and its adducts are found in a 21-amino-acid peptide [48,49]. This peptide, which Rappaport’s group has referred to as ‘T3’ (i.e., the third-heaviest tryptic peptide [49]), has been used as an analyte in a number of studies [49,50,51]. When a combination of trypsin and chymotrypsin is used, the Cys-34-containing peptide is instead the LQQCPF hexapeptide [43]. The use of Pronase, suggested by Sabbioni and Turesky as a means of generating lower-molecular-weight analytes, has not to our knowledge been implemented for untargeted HSA Cys-34 adductomics [36]. When Noort et al. [44,52] used Pronase to digest HSA adducts of either sulphur mustard or acrylamide, the respective modifications were found in the CPF tripeptide. 

Some of the first untargeted HSA adductomic analyses were performed by Aldini et al. using the technique of precursor ion scanning [43] (see also the ‘chemical modificomics’ method proposed by Goto et al. [53]). Precursor ion scanning is an MS/MS technique involving a scan at the first stage of mass analysis and the isolation of a constant product ion at the second stage. The result is a spectrum of the different precursor ions that give rise to a given product. Aldini et al. [43] reacted purified HSA with a mixture of α,β-unsaturated aldehydes (4-hydroxy-2-nonenal, 4-hydroxy-2-hexenal and acrolein), and digested the products with trypsin and chymotrypsin. Analysis of the digestion products, using liquid chromatography (LC) and online precursor ion scanning, revealed peaks corresponding to substituted LQQCPF peptides. These, in turn, corresponded to HSA Cys-34 Michael adducts of the α,β-unsaturated aldehyde reactants.

### 2.8. Fixed-Step SRM of HSA Adducts

An important development, reported by Li et al. in 2011, was the demonstration of a stepped method called fixed-step SRM (FS-SRM [49]). FS-SRM consists of a sequence of SRM experiments that collectively resemble a linked scan [54]. In developing the method, Li et al. drew on elements of the ‘adductome approach to detect DNA damage’ described by Kanaly et al. [34,55], and also a method of analysing mercapturic acids described by Wagner et al. [56]. Being a stepped method, FS-SRM is broadly analogous to the FI*R*E screening procedure (which, in fact, it pre-dates). The analytes in FS-SRM are substituted T3 peptides, and the precursor ions captured in the first stage of mass analysis are triply-protonated peptides. The product ions isolated in the second stage are doubly-charged variable *y*-ions and a singly-charged constant *b*-ion. Together, these precursor and product ions constitute what is effectively a peptide sequence tag [57]. The sampling points used for FS-SRM are 4.5 Da apart and, in the Li and co-workers’ study, there were 77 of them. FS-SRM differs from the other stepped methods in that, for FS-SRM, the sample is infused into the mass spectrometer as a mixture of adducts rather than as a series of eluted components. There is still an LC step but it is disconnected from the mass spectrometry, and it serves to capture the entire population of adducts rather than to separate them. The method is therefore freed from a major constraint imposed by LC, namely the need for a full set of SRM experiments to be done within the width of a chromatographic peak.

Our personal experience with protein adductomics has been in the implementation of FS-SRM for epidemiological studies [10,33]. Such studies, which typically involve tens or hundreds of samples, pose challenges that are not necessarily encountered in smaller pilot studies. In implementing the method of Li et al., the main challenge that we faced was the need for higher throughput. This was addressed by evaluating the various stages of sample preparation (HSA purification, adduct enrichment, digestion and peptide clean-up) and optimising these where possible. Notably, we deleted the adduct enrichment step, and we changed the method of sample clean-up from HPLC (serial) to solid-phase extraction (SPE; effectively parallel). A model adduct, prepared by treating HSA with *N*-ethylmaleimide, proved useful for evaluating the performance of the methods. 

In parallel with our work on FS-SRM, Grigoryan et al. [50] developed a new analytical workflow based on LC with on-line DDA mass spectrometry. In DDA, the *data* on which the acquisition is *dependent* are precursor ions’ *m*/*z* values, and they are obtained via a high-resolution scan—using, for example, an Orbitrap mass analyser. The *data* are used to direct the isolation of precursor ions, and so only these precursor ions are fragmented. The *acquisition* is the scan via which the resulting product ions are detected. In addition to their analytical method, Grigoryan et al. [50] also developed methods for sample preparation and data analysis (the ‘adductomics pipeline’). The method of sample preparation is essentially a streamlined version of the one developed by Li et al. [49]. One major difference with respect to the earlier method, however, was the omission of a reducing agent, which had previously been used to reduce protein disulphide bonds prior to tryptic digestion. The effect of omitting the reducing agent was to preserve *S*-thiolated forms of Cys-34. The method of data analysis begins with the detection of a tag (a combination of constant and variable product ions) in the product-ion scan data. The corresponding precursor ion is then identified, and an ion count chromatogram for this precursor ion is extracted. A particularly innovative part of the pipeline is the method by which the peptide analytes are quantified. Each analyte is quantified relative to a ‘housekeeping peptide’, which is another tryptic peptide of HSA. In this way, the method is able to control for variation in the quantity of digested HSA. Grigoryan et al. [50] used their pipeline to analyse samples of plasma from smokers and non-smokers, and found a total of 43 putative adducts (see Section 3 below).

### 2.9. Multiplex Adduct Peptide Profiling

Another promising method for HSA Cys-34 adductomics (and potentially also Hb Cys-β93 adductomics) is ‘multiplex adduct peptide profiling’ (MAPP [51]). MAPP utilises DIA mass spectrometry, which is perhaps the least prescriptive of all MS/MS techniques. Similar to a stepped SRM-based method, DIA captures precursor ions via a series of contiguous windows. The windows are, however, rather wider than those used for SRM, and it is therefore likely that a given window will capture multiple precursor ions (in MAPP, for example, the width of each window is 10 *m/z* units). As in DDA mass spectrometry, the second stage of mass analysis is a scan, and a high-resolution scan is done as an alternative first stage.

The MAPP method, like the ‘adductomics pipeline’, requires prior knowledge of the peptide analyte’s sequence and the site of modification. Series of constant product ions (e.g., b-ions from backbone scission near the N-terminus) are recognised and are linked back to their respective precursor ions via common chromatographic retention times. The substituted peptide’s mass shift is then confirmed by the presence of corresponding variable product ions. Although the authors were only able to identify oxidised and *S*-thiolated forms of HSA Cys-34, their method has the potential to detect toxicologically-relevant adducts (e.g., if the samples could be further enriched for these adducts prior to analysis).

### 2.10. Hb and HSA Compared

Given that Hb and HSA contain some of the same nucleophilic functional groups, these proteins might be expected to have overlapping reactivity towards electrophiles. The observation that cysteine residues in HSA and Hb can add to comparable amounts of benzene oxide in vivo, for example, is evidence of such overlap [58]. On the other hand, Dingley et al. [59] found that dietary exposure to 2-amino-1-methyl-6-phenylimidazo[4,5-*b*]pyridine (PhIP; see Section 3.3) caused the formation of substantially larger amounts of HSA adducts than Hb adducts. A similar fate has been observed for aflatoxin B_1_ in rats: of a given dose of this toxicant, a substantially higher proportion is found bound to serum albumin than to Hb [60,61]. This might also be expected to be the case in humans, and indeed assays for HSA adducts of aflatoxin B_1_ dialdehyde have been developed [62]. Possible reasons for differences in the amount or type of adducts include (i) the fact that Hb and HSA are synthesised at different sites in the body (in different cell types), and as a result could be exposed to different electrophiles [36]; (ii) the fact that Hb resides inside the erythrocyte, whereas HSA is secreted [18]; (iii) the influence of neighbouring amino acid side chains and cofactors on the reactivity of the nucleophilic groups (see Section 2.1 and Section 2.6); and (iv) the possibility that the erythrocyte membrane could shield Hb from electrophiles, or even sequester electrophiles [63]. It is also worth considering that apparent differences in the extent of adduct formation could reflect differences in chemical and biological stability of the proteins and/or modifications.

### 2.11. Other Target Proteins

Few proteins other than Hb and HSA have been discussed as candidates for untargeted adductomic analyses, and fewer still have been investigated experimentally. Hb and HSA adducts are probably two of the richest and most accessible sources of potential biomarkers, but this is not to say that other proteins could not provide additional and unique information. Three other proteins of relevance to the present review have been discussed: Collagen, histones and apolipoproteins. Collagen is mentioned by Scheepers in his workshop report [19], presumably because of its abundance in the body and its extremely long lifespan in certain tissues [64]. However, there have been few attempts to use collagen adducts for biological monitoring, probably because of the heterogeneity, physical properties and limited accessibility of collagen [65,66,67]. Histones, which are also mentioned by Scheepers, represent a more promising source of biomarkers. Work on histone adducts has not been extensive, but some interesting results have been obtained. *N*-Terminal segments of histones are of particular interest because they protrude from nucleosomal core particles, and, on this basis, it is plausible that they could be accessible to electrophiles. Consistent with this idea, SooHoo et al. [68] observed modifications near the N-termini of histones isolated from cultured human lymphoblasts that had been exposed to *anti*-benzo[a]pyrene 7,8-dihydrodiol-9,10-oxide (BPDE). Fabrizi et al. [69] used a model peptide to infer the reactivity of an *N*-terminal segment of histone H2B towards phosgene, and observed the incorporation of carbonyl groups into the peptide.

Apolipoproteins have been investigated as targets of endogenous electrophiles, such as the lipid oxidation product 4-hydroxy-2-nonenal. By definition, endogenous adducts cannot be biomarkers of exposure in the strict sense, but they could potentially be biomarkers of effect. We mention them here because they have been the subject of a recent untargeted adductomics study. This study focused on adducts of histidine and lysine residues in human low density lipoprotein [35]. Unlike the FI*R*E screening procedure or FS-SRM, the method is not site-specific; rather, it detects modifications to any and all residues of particular amino acid. The analytes are ‘free’ amino acids, which are prepared from lipoprotein by acid hydrolysis. Consequently, they may represent a mixture of sites, and perhaps a mixture of proteins. The analytical method, like others described elsewhere in this article, involves ultraperformance LC and triple quadrupole mass spectrometry. Apparently it is a stepped method, in which a constant product ion is isolated at the second stage of mass analysis. For adducts of histidine residues, the constant product is the immonium ion of histidine, and for adducts of lysine residues, it is a deaminated immonium ion of lysine. Shibata et al. [35] used their method to analyse low density lipoprotein that had been first purified from human plasma, and then oxidised in vitro. The oxidised lipoprotein was treated with sodium borohydride to reduce imine linkages (as in, for example, a lysine residue adducts of 9-oxononanoic acid), before being hydrolysed and the resulting amino acids analysed. The authors produced adductome maps for lipoprotein with and without oxidation, and by comparing these maps they were able to attribute the formation of the aforementioned 9-oxononanoic acid adduct to the oxidising condition.

### 2.12. Adduct Enrichment

Enrichment, in the context of untargeted adductomics, entails depletion of the unmodified nucleophile and possibly also other substances that might interfere with the detection of the adducts. In the FI*R*E screening procedure for Hb adducts, enrichment is facilitated by the detachment of the *N*-alkylvaline residues. This exaggerates the relatively minor difference in structure between Hb and its adducts, thereby allowing the unmodified Hb to be removed readily [28]. For HSA Cys-34 adductomics, methods of enrichment have mainly exploited the reactivity of the Cys-34 thiol group, which is present in the unmodified HSA but not in the adducts. Funk et al. [70] demonstrated the use of a disulfide-functionalised resin for scavenging unmodified HSA, and this method was later used in adductomic workflows [49,51,71]. The main limitation of the thiol scavenging method is that it does not remove *S*-thiolated HSA: If a reducing agent is later added to reduce the other disulfide bonds in HSA (i.e., those of the cystine residues) then the *S*-thiolation is reversed and the Cys-34 thiol would seem to reappear. Funk et al. [70] sought to limit this effect by removing the S-thiolation prior to the scavenging step. In our hands, the thiol scavenging method proved difficult to implement in a high-throughput setting, and so we deleted it from our workflow [33]. Chung et al. [71] used thiol scavenging as the first of two stages of enrichment, the second stage being an antibody-mediated purification of the substituted T3 peptides using a polyclonal antibody raised against the T3 peptide but having cross-reactivity with adducts.

## 3. Human Biomonitoring

### 3.1. Methodological Considerations

Human biomonitoring refers to the quantification of xenobiotics or their derivatives (and sometimes their early effects) in human biospecimens [72]. As well as confirming the nature of the exposure, biomonitoring aims to measure the internal dose of the xenobiotic(s). The biomonitoring of protein adducts is usually done as part of the ‘bottom up’ (targeted) approach (see Section 1). A typical targeted method might involve isotope dilution (i.e., the addition of a known amount of an isotopically-labelled standard) followed by LC-MS/MS. This would require prior characterisation of the adduct and synthesis of a suitable standard.

In principle, data collected via the untargeted approach (e.g., peak areas from LC-MS/MS) could be used in the same way as those collected in targeted studies. However, this would depend on the untargeted method achieving an acceptable accuracy, precision and dynamic range for each relevant adduct. At some stage, a synthetic reference compound would be needed to confirm a particular adduct’s identity, and to implicate the corresponding electrophile [18]. For hitherto unknown adducts, possible identities must first be proposed. Methods that have assisted in this endeavour have included database searching, the use of calculator software, and the comparison of measured and predicted physicochemical properties [50,73]. The characterisation of novel adducts—a challenging aspect of the research—has been reviewed in detail by Carlsson et al. [18].

Accuracy, in practice, may suffer as a consequence of the need to capture a range of adducts. It is likely that the use of generic standards (e.g., the S-carbamidomethylated T3 peptide for FS-SRM) affects accuracy, and therefore precludes absolute quantification [33]. Dynamic ranges are dependent on the analytical method, and presumably also on the ability to enrich adducts. As judged from lowest reported adduct concentrations, the detection limits of Grigoryan and co-workers’ LC-MS-based method, and of the FI*R*E screening procedure, are good (<7 and <0.1 adduct molecules per million HSA molecules or Hb chains, respectively [28,50]). The methods should, therefore, be able to detect some xenobiotic adducts, although in practice relatively few such adducts have been observed [50]. For FS-SRM (our implementation), the detection and quantification limits are in the region of one adduct molecule per thousand HSA molecules, and are probably too high to detect xenobiotic adducts [33]. Putative adducts detected by FS-SRM and the other methods may, however, relate to the early effects of exposure.

At the present time, the role of the untargeted methods is to complement the targeted methods, rather than to replace them. Indeed, approaches that combine both methods have been proposed [7]. Some authors advocate a more pragmatic ‘fit-for-purpose’ approach, which balances methodological rigour with cost. Dennis et al. draw a distinction between regulatory endeavours, which require maximal rigour, and exploratory studies whose aims might be achievable without a fully validated method [7].

While the discipline of untargeted protein adductomics is still a relatively young one, there have been a number of pilot studies that have sought to demonstrate its utility. Additionally, some targeted investigations have looked for adduct formation at the same sites (e.g., Cys-34 of HSA) and these will also be mentioned here.

### 3.2. Human Biomonitoring of Hb Adducts 

In the first adductomic application of the FI*R*E method (see Section 2.5), Hb samples from smokers and non-smokers were analysed and compared [28]. In all samples seven adducts at the *N*-terminal valine residue were identified; these were the addition of methyl and ethyl groups, and adducts formed by ethylene oxide, acrylonitrile, methyl vinyl ketone, acrylamide and glycidamide; in addition, a further 19 unknown adducts were detected in all samples. Subsequently, one of these unknown adducts has been identified as derived from ethyl methyl ketone [74]. A further four have been attributed to the precursor electrophiles glyoxal, methylglyoxal, acrylic acid and 1-octen-3-one [73]; and recently another adduct, detected in smokers and non-smokers at similar levels, has been identified as *N*-(4-hydroxybenzyl)valine, postulated to have arisen from either 4-quinone methide, which could form the valine adduct via a Michael addition, or 4-hydroxybenzaldehyde, which could form the same adduct via a Schiff base formation followed by reduction [75].

Applying their untargeted Hb adductomic approach to a larger study population, Carlsson et al. [76] analysed blood samples from healthy children about 12 years old (*n* = 51). In this cohort, a total of 24 adducts (12 of them previously identified; see above) were observed and their levels quantified. Relatively large interindividual variations in adduct levels were observed. The frequencies of micronuclei in erythrocytes were also determined. Analysis using a partial least-squares regression model showed that as much as 60% of the micronucleus variation could be explained by the adduct levels. This indicates the ability of such studies to align measurements of internal dose (protein adducts) with endpoints of genotoxicity (micronucleus formation). 

### 3.3. Human Biomonitoring of HSA Adducts 

An early study that demonstrated the utility of monitoring HSA for alkylated cysteine involved exposure of human blood to ^14^C-labelled sulfur mustard (the chemical warfare agent mustard gas) [44]. Isolation and tryptic digestion of albumin produced the 21-amino acid fragment containing a sulfur mustard-cysteine adduct, detected by micro-LC-MS/MS. An alternative method, which employed Pronase for the digestion, yielded a modified tripeptide (Cys-Pro-Phe), which was detected with greater sensitivity than the 21-amino acid fragment. The method was used to analyse samples of blood from nine Iranians exposed to sulfur mustard during the Iran-Iraq war of 1986. In all nine cases, the sulfur mustard-adducted tripeptide was detected.

Application of the FS-SRM method to analyses of archived plasma protein that had been pooled according to subjects’ ethnicities and tobacco smoking habits demonstrated differences between pools [49] and suggested that FS-SRM might be able to detect statistically significant differences between groups of individual samples that had not been pooled.

A pilot study of 20 smokers and 20 never-smokers provided evidence of the effect of smoking on levels of putative HSA adducts. Differences between smokers and never-smokers were most apparent in putative adducts with net gains in mass between 105 Da and 114 Da (relative to unmodified HSA) [33].

Further investigations of the effects of tobacco smoking have revealed around 43 adduct features, some of which are positively associated with smoking and but also some that are negatively associated. The former result from genotoxic constituents of tobacco smoke, such as ethylene oxide and acrylonitrile, while the latter, which include Cys-34 oxidation products and disulfides, may reflect alterations in the serum redox state of smokers, resulting in lower adduct levels [50]. 

Grigoryan et al. used LC and high-resolution mass spectrometry to investigate interactions between the Cys-34 and reactive oxygen species (ROS) [47]. Chronic exposure to ROS is linked to many chronic diseases and, in this study, a number of adducts originating from ROS were detected in human serum: Sulfinic acid, sulfonic acid and a proposed sulfinamide structure (a mono-oxygenated moiety also with the loss of two hydrogen atoms).

Antibody enrichment may pave the way to a more sensitive assay. Using a polyclonal antibody, raised against the T3 peptide, but with cross-reactivity to the peptide containing adducts (see Section 2.12), ten modified T3 peptides were detected in human plasma samples; eight of them were characterised and they included Cys-34 oxidation products, modification involving loss of water or lysine, cysteinylation, and transpeptidation of arginine [71].

In a study of women from the Xuanwei and Fuyuan counties in China, where extensive use of smoky coal for heating and cooking has resulted in very high rates of lung cancer among non-smokers, HSA Cys-34 adducts were compared in 29 females who used smoky coal and 10 controls using other energy sources [77]. Fifty different modified T3 peptides were identified, including oxidation products, mixed disulfides, rearrangements and truncations. Two peptides that were detected at significantly *lower* levels in the smoky coal group were adducts of glutathione and γ-glutamylcysteine. The results are interpreted as evidence that exposure to the indoor combustion products results in depletion of glutathione, an essential antioxidant, as well as its precursor γ-glutamylcysteine [77].

A recent study on the health effects of urban air pollution, the Oxford Street II study [78], involved a randomised crossover design whereby three groups of volunteers (healthy subjects, chronic obstructive pulmonary disease (COPD) sufferers and patients with ischaemic heart disease (IHD)) walked for two hours along a busy street in London where traffic is restricted to diesel buses and taxis. The volunteers also spent two hours walking in a London park on a separate occasion. They were monitored for respiratory and cardiovascular function in both environments and, in addition, two studies have analysed their HSA samples for adducts. In the first report, Liu et al. [79] analysed 50 HSA samples by high-resolution mass spectrometry to determine whether protein modifications differ between COPD or IHD patients and healthy subjects. The untargeted analysis of adducts at the Cys-34 locus of HSA detected 39 adducts with sufficient data, and these adducts were examined for associations with estimated exposures to air pollution and health status. Multivariate linear regression revealed 21 significant associations, mainly with the underlying diseases, but also with air-pollution exposures. Interestingly, most of the associations indicated that adduct levels decreased with the presence of disease or increased pollutant concentrations. Negative associations of COPD and IHD with the Cys-34 disulfide of glutathione and two Cys-34 sulfoxidations were consistent with results from smokers and non-smokers [50] and from non-smoking women exposed to indoor combustion of coal and wood [77].

In the second study, Preston et al. [80] examined a larger number of Oxford Street II samples by the FS-SRM method. Associations between amounts of putative adducts and two types of measure were tested: Pollution (e.g., ambient concentrations of nitrogen dioxide and particulate matter) and health outcome (e.g., measures of lung health and arterial stiffness). There were 11 instances of a response variable being associated with a pollution measurement and eight instances of a response variable being associated with a health outcome measure. However, no two measures of different types were associated with the same adduct amount, suggesting that the internal changes responsible for health outcomes may differ from those that effect changes in adduct amounts. 

In a more targeted study, Bellamri et al. [81] investigated the formation in human subjects of HSA adducts at Cys-34 by PhIP, which is formed in cooked meats and may be associated with colorectal, prostate and mammary cancer. Volunteers abstained from eating cooked well-done meat or fish for three weeks, then ate a semi-controlled diet that included cooked beef containing known quantities of PhIP for four weeks. The volunteers then returned to their regular diets, but with the exclusion of cooked well-done meat and fish for a further four weeks. The authors found that an adduct of oxidised PhIP, which was below the limit of detection (LOD) (10 femtograms PhIP/mg HSA) in most subjects before the meat feeding, increased by up to 560-fold at week 4 in subjects who ate meat containing 8.0 to 11.7 μg of PhIP per 150–200 g serving. In contrast, the adduct remained below the LOD in subjects who ingested 1.2 or 3.0 μg PhIP per serving, and PhIP-HSA adduct levels did not correlate with PhIP intake levels across four exposure groups (*p* = 0.76). There were also indications that the PhIP adduct was unstable, having a half-life of fewer than two weeks. Nevertheless, the study demonstrates that the Cys-34 site in HSA is accessible by a relatively large molecule like PhIP, despite concerns about possible steric hindrance (see Section 2.6.).

## 4. Prospects

A key advantage of monitoring proteins for adducts is the abundance of material that can be obtained from tissue banks; for example, red blood cells are an abundant source of Hb and blood plasma or serum is an abundant source of HSA. The proteins’ lifespans in blood mean that there is a substantial “capture period” for monitoring exposure to genotoxicants; and protein adducts, unlike DNA adducts, are not subject to loss through repair processes. Full implementation of the exposome concept requires monitoring individuals or populations at several points in time over the course of their lives [3,4]. This is achievable if biobanks collect material from individuals not just once but multiple times, and such biobanks already exist. 

Dried blood spots can also be a suitable source of protein for investigation [82]. If obtained from neonatal blood spots (i.e., Guthrie spots) then the material provides a valuable opportunity for investigating exposures in utero. A single blood spot of about 50 μL is estimated to contain about 9.6 mg of protein, of which about 7.7 mg will be Hb and 1.2 mg HSA [82]. In a proof-of-principle study, Yano et al. [83] identified 26 Cys-34 adducts (oxidation and *S*-thiolation products) in HSA isolated from dried blood spots of 49 newborn babies and were able to distinguish between newborns of smoking and non-smoking mothers on the basis of the levels of a putative cyano modification to Cys-34.

There is also the potential to broaden the scope of adductomics by investigating novel modifiable loci in blood proteins. Cys-34 of HSA and the *N*-terminus of Hb are undoubtedly major targets for electrophiles, but the literature hints at wider reactivity within the blood proteome. Consequently, a hitherto-untapped source of analytes for protein adductomics can be envisaged. Mapping the loci at which adducts can form will be beneficial and, for this purpose, new chemical tools will be required. Identifying adducts detected by the top-down approaches will be a challenge, necessitating chemical synthesis of candidate structures for unequivocal characterisation.

Protein adductomics is a component of the exposome concept that is still relatively novel, but it is one that has already demonstrated the ability to capture electrophiles of both endogenous and exogenous origin; this suggests the potential to contribute meaningfully to the aims of the exposome concept—to describe the totality of all biologically relevant exposures. Rapid advances in mass spectrometry instrumentation, with significant increases in sensitivity and resolution, will drive further advances in protein adductomics methodology. When coupled with other omics approaches, such as proteomics, transcriptomics and metabolomics, all of which have the potential for high-throughput screening of populations, a future can be envisaged in which it will be possible to capture snapshots of human exposure to genotoxicants and the resultant biological consequences at multiple stages throughout life. Building this comprehensive picture should shed significant light on the causes and courses of chronic diseases in humans. Such knowledge will provide new opportunities for early intervention to reduce potentially harmful human exposure, to monitor the effectiveness of intervention strategies and, ultimately, to prevent diseases before they occur.

## Footnotes

1 A note regarding language. For the reaction of a nucleophile with an electrophile, the view of the chemist is that the *nucleophile* is the active participant, providing electrons for the chemical bond (‘nucleophilic addition’, ‘nucleophilic attack’, and so on). Toxicologists, on the other hand, tend to speak of the *toxicant* as active (toxicant ‘binds’ to target), and since the toxicant is usually an electrophile the roles would seem to switch. This second interpretation is equally logical because the nucleophilic targets are often endogenous and less mobile (e.g., DNA or protein) and therefore seem to be passive entities.
